# Timeliness of childhood vaccine uptake among children attending a tertiary health service facility-based immunisation clinic in Ghana

**DOI:** 10.1186/1471-2458-14-90

**Published:** 2014-01-29

**Authors:** Dennis Odai Laryea, Emmanuel Abbeyquaye Parbie, Ebenezer Frimpong

**Affiliations:** 1 Public Health Unit, Komfo Anokye Teaching Hospital, P.O. Box 1934, Kumasi, Ghana; 2 Department of Paediatrics, 37 Military Hospital, Neghelli Barracks, Accra, Ghana; 3 National AIDS/STI Control Programme, Komfo Anokye Teaching Hospital, Kumasi, Ghana

## Abstract

**Background:**

Childhood immunisation is a cost-effective activity in health. Immunisation of children has contributed to reducing child morbidity and mortality. In the last two decades, global deaths from vaccine-preventable illnesses have decreased significantly as a result of immunisation. Similar trends have been observed in Ghana following the introduction of the Expanded Programme on Immunisation. The administration of vaccines is based on the period of highest susceptibility among others. Ghana has long used the proportion of children receiving vaccines and the trends in vaccine preventable illness incidence as performance indicators for immunisation. The addition of timeliness of vaccine uptake as an additional performance indicator has been recommended. This study evaluated the timeliness of vaccine uptake among children immunised at the Komfo Anokye Teaching Hospital, Kumasi, Ghana.

**Methods:**

The study was conducted at the Maternal and Child Health clinic of the hospital between February and March 2012. A representative sample of 259 respondents was selected by simple random sampling. Data collection was by a structured questionnaire and included the examination of Child Health records booklet. Data was entered into a Microsoft Office Access database and analysed using Epi Info Version 3.5.1 2008.

**Results:**

The majority of mothers attended antenatal clinics during pregnancy. An overwhelming majority of babies (98.8%) were delivered in a hospital. About 85% of babies were less than 12 months of age. Mean time taken to reach the clinic was 30 minutes. Vaccine uptake was generally timely for initial vaccines. The proportion of children receiving the vaccines later increased with latter vaccines. Overall, 87.3% of babies received vaccines on time with only 5.3% receiving vaccines beyond 28 days of the scheduled date. Children receiving immunisations services in the same facility as they were born were more likely to receive the BCG vaccine on time.

**Conclusions:**

Vaccine uptake is mostly timely among respondents in the study. The BCG vaccine in particular was received on time among children born in the same facility as the immunisation clinic. There is the need to further examine the timeliness of vaccine uptake among children delivered outside health facilities in Ghana.

## Background

Childhood immunisation has been identified as one of the most cost effective interventions in healthcare delivery [1,2]. Significant gains have been made through immunisation resulting in a reduction in the burden of vaccine preventable illness globally with an estimated 35 million dollars to be spent between 2006 and 2015 on vaccines [3]. In Ghana, immunisation has been a core public health activity through the Expanded Programme on Immunisation (EPI) since1985 [4]. The current EPI schedule recommends Bacille Calmette-Guerin (BCG) at birth; a total of 4 doses of Oral Polio Vaccine (OPV) given at birth, 6, 10 and 14 weeks of age (OPV 0, OPV1, OPV 2 and OPV 3 respectively); 3 doses of Diphtheria, Pertusis, Tetanus, Haemophilus influenzae type B and Hepatitis B (DPT/HiB/HepB) 5-in-1 vaccine at 6, 10 and 14 weeks of age. Measles and Yellow fever vaccines are administered at 9 months of age [5]. Ghana’s schedule differs from other countries [6] such as Nigeria where for example the Hepatitis B vaccine is given at birth. Immunisation aims at reducing disease burden and associated mortality. Ghana’s high under-5 mortality rate [7] coupled with the non-availability of skilled healthcare professionals and health facilities [8] make immunisation an essential health intervention.

Vaccine administration at recommended age is important as such recommendations are based on the estimation of the age at which a child’s risk for target diseases is highest [9]. The Measles vaccine for example is administered earlier, usually by 9 months of age, in most developing countries such as Ghana and Nigeria [5,6,10] because of the higher risks of transmission of the disease compared with developed countries as England [11] where the vaccine is given later. Timely receipt of vaccines is important because it ensures that the recipient is protected from target diseases as early as possible. Delayed administration of vaccines can result in longer periods of susceptibility among children and the presence of such a pool of such susceptible children can result in an epidemic when a case of a specific vaccine-preventable illness occurs [10]. The timely administration of vaccines as recommended has been found to be uncommon [12] and this may present a challenge to achieving the core objective of vaccination programmes which is to prevent diseases from occurring. Performance indicators for Ghana’s EPI have focussed on the proportion of children receiving vaccines and reported cases of vaccine-preventable illnesses [4]. The timeliness of vaccine administration has been recommended for inclusion along with other measures such as age-appropriate coverage in evaluating the quality of vaccination programmes [9,12-14]. Delayed vaccine uptake may have implications for public health programmes including the occurrence of fatal disease in individuals, outbreaks, and negatively impact national and international targets of disease elimination [15].

Delayed uptake of vaccines may be due to cultural factors, poor supervision of health workers and poor programme planning and monitoring and increasing age of infant [14,16]. Individual and community based factors including religion as well as immunisation services offered on outreach basis have been associated with an increased likelihood of delayed vaccination [17,18]. Hospital-based immunisation services have however been associated with delay in receipt of vaccines in Italy [19]. The advantage of maternal education in vaccine uptake has been discounted although the association between prenatal service and continuation of immunisation in the same facility has been associated with complete vaccination [20]. Hospital birth has been associated with timely uptake of vaccines [14,21] but Ghana has consistently recorded low rates of hospital delivery. Less than 50% of births are supervised by qualified health workers in health facilities despite high levels of antenatal care (ANC) attendance among pregnant women [7]. This study was conducted in light of the foregoing and the fact that no such examination of timeliness of vaccine uptake has been undertaken in Ghana.

## Methods

The study was conducted at the Komfo Anokye Teaching Hospital’s (KATH) Maternal and Child Health (MCH) clinic. It is one of several government health facilities providing immunisation services for pregnant women and children in Kumasi and surrounding communities. The clinic sees an average of 2000 children per annum and is open on Mondays to Fridays except on weekends and public holidays. The clinic provides immunisation services for all vaccines as per Ghana’s EPI schedule on days of activity and provides other services such as growth monitoring for children up to age 5.

The study design was a descriptive cross-sectional type and involved the use of a structured questionnaire. The questionnaire was pre-tested and some modifications made. A representative sample of 259 was used and was based on an estimation of a 75% timeliness of vaccine administration at 95% confidence interval and an estimated population of 2,000 using Epi Info Version 3.5.1.2008. The administration of the questionnaires was conducted in the two-month period February to March 2012. Written informed consent was obtained from all respondents after explanation of the purpose of the study including the voluntary nature of participation of potential respondents. Selection of subjects was by simple random sampling and was irrespective of the duration of clinic attendance. Random numbers were generated based on the chronology of entries in the clinic records for the twelve months prior to the start of data collection. Selected children were identified on the date of clinic attendance for the administration of the questionnaire. Records of previous attendance in the Child Health Records booklet and clinic records were examined as part of data collection. Details of demographic information, immunisation history and dates of administration, ANC and delivery histories were recorded. Maternal information, distance to health facility, antenatal records and delivery details were obtained from mothers or accompanying relations.

Ethical approval for the study was obtained from the KATH/Kwame Nkrumah University of Science and Technology Committee on Human Research and Publication Ethics.

Data was entered into an access database and analysed using Epi Info Version 3.5.1 2008. Tables and charts were created using Microsoft Excel. The timeliness of vaccine administration was determined based on the schedule of the Ghana’s National Immunisation Programme as follows for a specific vaccine as follows: Too early if the vaccine was received earlier than the recommended age; On time if the vaccine was received on or within two weeks after the due date; Acceptably early if received between two and four weeks after the due date, and Delayed if received after four weeks of the due date [10]. The timeliness of vaccine administration in days was calculated by subtracting the expected date of vaccine administration, based on the date of birth of the child, from the date of administration of the specific vaccine. Overall timeliness was calculated using the same criteria outlined above for all vaccines administered to children in this study.

## Results

### Demographic, antenatal, delivery and post natal characteristics

There were a total of 113 males and 146 (56.4%) females. The majority of babies 124 (76.1%) were 9 months of age or younger with 1.2% over 24 months of age. The mean age (SD) of children was 7.6 (4.8) months, median age 7 months and range of 2 to 28 months. The mean age (SD) of mothers was 30.8 (5.9) years with modal and median ages being 30 years and a range of 18 to 45 years. On education, 40% of mothers had at least secondary education with 9 representing 3.5% having had no formal education. ANC and delivery details are as shown in Table 1.

**Table 1:** ANC and delivery details for children seen at the KATH MCH clinic, 2012

**Parameter**	**Frequency**	**Percentage**
**Place of ANC**		
KATH	118	45.6
Other health facility	140	54.0
Not attended	1	0.4
**Total**	**259**	**100**
**Number of ANC visits**		
**0**	1	0.4
1 or 2	2	0.8
3 or 4	26	10.0
>4	230	88.8
**Total**	**259**	**100**
**Place of delivery**		
KATH	174	67.2
Other health facility	82	31.6
Home	3	1.2
**Total**	**259**	**100**
**Delivery by**		
Doctor	99	38.4
Midwife/Nurse	156	60.5
Other	4	1.1
**Total**	**258**	**100.0**
**Mode of delivery**		
Spontaneous vaginal	163	63.2
Caesarean section	90	34.9
Assisted vaginal	5	1.9
**Total**	**258**	**100.0**

Public transport was the most commonly used means of transport with an average time of 33 minutes taken to reach the clinic. The minimum and maximum times were 5 and 120 minutes respectively with median and modal times both being 30 minutes.

Only one infant’s mother did not attend ANC during pregnancy. Approximately 90% of mothers made more than 4 ANC visits. All but 3 (1.2%) babies were delivered in a health facility. The majority (67%) of infants were delivered in KATH. The majority of babies were delivered by midwives. Doctors delivered about 38% of babies by Caesarean Section (Table 1). The mode of delivery was unknown for one baby as the mother died after delivery. A total of 27 babies (10.4%) were said to be unwell after delivery and were admitted. Of the 27 babies admitted after delivery, 13 were delivered by Caesarean section and the remainder by spontaneous or assisted vaginal delivery. The majority of these babies were delivered in KATH (77.8%).

#### Vaccine uptake

All infants in the study had received BCG immunisation. The majority of babies received this vaccine within 2 weeks of delivery (88.8%). A total of 17 babies (6.6%) received the BCG vaccines after 28 days of age (‘Delayed’). Compared with children delivered in KATH, children delivered in other sites were less likely to receive the BCG vaccine on time (OR = 4.16, 95% CI: 1.48- 11.68, p = 0.004). A total of 239 babies had received the OPV 0 vaccine and 238 babies OPV 1 and DPT/HiB/HepB 1 vaccines. The majority received these vaccines on time (89.6%) with 2.7% receiving it more than 28 days after the due date. The timeliness of administration of OPV 2 and 3 and DPT/HiB/HepB 2 and 3 are shown in Table 2. The Measles and Yellow fever vaccines had the highest proportion of babies receiving the vaccines later.

**Table 2:** Timeliness of vaccine uptake for specific vaccines at the KATH MCH clinic, 2012

**Vaccine**	**Timeliness of receipt of vaccine**							
	**Too early**		**On time**		**Acceptably early**		**Delayed**	
	**No.**	**%**	**No.**	**%**	**No.**	**%**	**No.**	**%**
BCG*	N/A	N/A	230	88.9	12	4.6	17	6.6
OPV 0	N/A	N/A	229	95.8	10	4.2	0	0
OPV/DPT/HiB/HepB 1	6	2.3	232	89.6	14	5.4	7	2.7
OPV/DPT/HiB/HepB 2	6	2.5	205	86.5	17	7.2	9	3.8
OPV/DPT/HiB/HepB 3	4	1.9	176	83.1	16	7.5	16	7.5
Measles/Yellow fever	3	3.1	49	50.5	29	29.9	16	16.5

*BCG- Bacille Calmette-Guerin; N/A: not applicable; OPV- Oral Polio Vaccine; DPT- Diphtheria, Pertussis and Tetanus; HepB- Hepatitis B.

Among children delivered by caesarean section, BCG and OPV 0 were administered on time for 96.7% and 96.6% for respectively. A total of 1,304 vaccines had been administered to all children in the study. The proportion administered ‘on time’ was 87.3% and total of 69 (5.3%) of vaccines were administered beyond 28 days (delayed) of the scheduled age of administration. Only 1.7% of vaccines were administered earlier than the scheduled dates (Table 3).

**Table 3:** Timeliness of vaccine administration for all vaccines

**Timeliness**	**Number**	**%**
**Early**	22	1.7
**On time**	1139	87.3
**Acceptably early**	74	5.7
**Delayed**	69	5.3
**Total**	**1304**	**100.0**

## Discussion

An overwhelming majority of mothers attended ANC during their respective pregnancies. This is much higher than the national average of women accessing ANC services [7] with only 1.2% of babies delivered outside a health facility. Considering the fact that Kumasi is an urban area with the majority clients being resident in Kumasi, this is consistent with the high proportion of deliveries in urban centres being supervised [7]. Hospital delivery has been associated with timely vaccine uptake [21] although in this study, children delivered in health facilities other than KATH received some vaccines later and is consistent with similar findings in Burkina Faso [19]. Further evidence in this study is the observed association between delivery in KATH and receipt of immunisation services in the same facility. Despite this, some children delivered in KATH received the BCG and OPV 0 vaccines much later indicating missed opportunities for immunisation [10] and must be critically examined.

Generally, the proportion of infants receiving vaccines on time decreased as the immunisation schedule progressed with BCG being the only deviation from this trend. This is consistent with finding of delayed uptake of vaccine with increasing age as observed by Fadness et al. [16]. It would have been expected that almost all the babies should have received BCG on time since they were born in a health facility. This seeming missed immunization opportunities particularly for other health facilities may be due to the fact that BCG unlike OPV 0 was given irrespective of how late the child was brought to the clinic. BCG is also not readily administered in some facilities as the convention is to get a minimum number of infants in order to reduce wastage of vaccines following its opening. There is also the possibility of time of delivery and /or discharge being responsible for missed opportunity for immunisation after delivery particularly if the delivery occurs on a weekend or in the night. Night deliveries may result babies discharged early in the morning with the possibility of missing the vaccination. Similarly, public health staffs responsible for the administration of the vaccine are not usually available on weekends and may account for some cases of missed opportunity for BCG as well. In the case of OPV 0, the requirement of a minimum of 4 weeks between doses meant some children missed the dose if they reported to the clinic after 2 weeks of delivery [5]. Vaccine uptake was extremely timely in this study compared with the one conducted in Nigeria [10]. This study found much higher levels of timely administration of vaccines with proportions ranging between 50.5% and 95.8% compared with 18.7 to 61.5% reported by Sadoh and Eregie [10]. Even higher proportions of children received vaccines more than 28 days after the due date with proportions ranging between 18.9 and 65% compared with this study’s finding of between 0 and 16.5%. They however did not include the place of delivery in their study as shown in a study in Nigeria [20] and may be a factor in the observed difference.

This study also found that the majority of babies delivered by caesarean section received the first vaccines on time. This finding is consistent with the association of hospital delivery with timely vaccine administration [21]. This study cannot determine the role of hospital birth in the timely administration of latter vaccines in the EPI schedule as these may be influenced by other factors. There may be the need to specifically examine the timeliness of vaccine uptake among children delivered outside hospitals to determine any relationship if it exists [19]. The fact that the majority of children in this study were delivered in a health facility is a marked deviation from national trends [7] and the results may therefore not be applicable to children delivered outside health facilities.

The proportion of children receiving vaccines on time was found to be lower among later vaccines and is consistent with findings by Fadness et al. [16] observed in South Africa. The observed increasing trend of the proportion of children receiving latter vaccines later than 28 days could be due to forgetfulness or the perception of irrelevance of clinic attendance following the receipt of the third dose of the 5-in-1 vaccine as clinic attendance then is only characterised by the weighing of children and no vaccine administration. In addition, the maternity leave for eligible women is only three months for most institutions in Ghana hence mothers may be working at these times and may be a factor in the delayed attendance of immunisation clinics. The reasons for this observation may require further investigation.

## Conclusion

A high proportion of children seen at the KATH MCH clinic received all vaccines on time with the proportion decreasing with increasing age of expected immunisation. A significant number of missed opportunities for immunisation still occur. Children who received immunisation services in the same facility as they were born appear to be more likely to receive the BCG vaccine on time.

The Ghana Health Service must devise means of ensuring that children delivered outside major health facilities receive vaccines on time. There will also be the need for further studies on the timeliness of vaccine uptake among children delivered outside health facilities. An examination of factors contributing to delayed uptake of later vaccines is also recommended.

## Competing interests

The authors declare that they have no competing interests.

## Authors’ contributions

DOL and EAP were both involved in the literature review and design of the study. DOL and EF analysed the data. EF was responsible for data collection. DOL did the write-up. DOL and EAP were responsible for proof-reading of the final manuscript. All authors read and approved the final manuscript.

## Authors’ information

Dennis Odai Laryea (DOL) is a Specialist Public Health Physician and Head of the Public Health Unit of the Komfo Anokye Teaching Hospital in Kumasi, Ghana. His special interests are in Reproductive and Child Health and activities aimed at achieving Millennium Goals 4, 5 and 6. He is a graduate of Medicine from the Kwame Nkrumah University of Science and Technology and holds and MSc in Public Health from the University of the West of England. He also holds a Membership in Public Health (MGCP) from the Ghana College of Physicians and Surgeons.

Emmanuel Abbeyquaye Parbie (EAP) is a Specialist Paediatrician and Head of the Neonatal Intensive Care Unit of the 37 Military Hospital in Accra, Ghana. He has special interests in Neonatology and Infectious Diseases. A graduate of Medicine from the Kwame Nkrumah University of Science and Technology, He holds membership in Paediatrics in both the Ghana College of Physicians and Surgeons and the West African College of Physicians. He is currently pursuing a Diploma in Project Design and Management (DPDM) with the University of Liverpool.

Ebenezer Frimpong (EF) is a Public Health Practitioner with a Degree in Social Sciences from the University of Ghana. He has been in Public Health practice since 2007 focussing on health promotion activities including HIV prevention.

## Pre-publication history

The pre-publication history for this paper can be accessed here:

http://www.biomedcentral.com/1471-2458/14/90/prepub
